# Toward embodied artificial cognition: TIME is on my side

**DOI:** 10.3389/fnbot.2014.00025

**Published:** 2014-12-08

**Authors:** Michail Maniadakis, Marc Wittmann, Sylvie Droit-Volet, Yoonsuck Choe

**Affiliations:** ^1^Foundation for Research and Technology - HellasHeraklion, Greece; ^2^Institute for Frontier Areas of Psychology and Mental HealthFreiburg, Germany; ^3^Laboratoire de Psychologie Sociale et Cognitive, CNRS (UMR 6024), Department of Psychology, Université Blaise PascalClermont-Ferrand, France; ^4^Department of Computer Science and Engineering, Texas A&M UniversityAustin, TX, USA

**Keywords:** time perception, embodied cognition, robotics, psychology, neuroscience

## Introduction

From the moment of birth, humans and animals are immersed in time: all experiences and actions evolve in time and are dynamically structured. The perception of time is thus a capacity indispensable for the control of perception, cognition and action. The last 10 years have witnessed a remarkable resurgence of interest in timing and time perception, with a continuously increasing number of researchers exploring these temporal abilities. However, existing robotic systems largely neglect the key role of time in cognition and action. This is a major barrier for accomplishing the long-term goal of symbiotic human-robot interaction. The critical question is: how is time instantiated in a biological system and how can it be implemented in an artificial system? Recent years have seen an increasing focus on the relationship between embodiment, affective states and the subjective experience of time. In particular, the influence of affective states on subjective time seems to depend on the embodiment of emotions. As a result, intertwined affective and interoceptive states probably contribute to our subjective experience of time. Since robotic systems are in essence embodied information-processing systems that interact with the real world, we hope to inspire a reciprocal exchange of ideas between the field of Robotics, Cognitive Neurosciences, and Psychology. The present Research Topic aims at paving the way for a new generation of intelligent computational systems that incorporate the sense of time in their processing loop and thus accomplish more efficient and more advanced cognitive capacities. Along this line, the development of robots which can sense the passage of time will significantly improve human-robot interaction particularly in natural tasks that require knowledge of and sensitivity to temporal factors.

In this research topic, we have called researchers from different disciplines (Psychology, Robotics, and Neuroscience) to present their empirical work, their models or reviews on the question of how time judgments are instantiated in biological and artificial systems. Of particular interest were papers on time perception in humans and animals—with a focused interest on embodied time perception—as well as papers discussing the key role of time on different aspects of robotic cognition and the neural underpinnings of timing related phenomena.

In the following, we organized the contributing articles into three categories: (1) psychology and embodied cognition, (2) robotics, and (3) neural underpinnings. First of all, we want to specifically point to the contributing article (Maniadakis and Trahanias, 2014) which provides a broad theoretical overview on the multitude of aspects of how time affects cognition. We suggest consulting this article prior to delving into the other articles in this research topic.

## Psychology and embodied cognition

Most of the manuscripts in this research topic on the psychology of time present studies on how subjective duration dilates in emotional situations. To examine these emotion-related time distortions, different emotional stimuli were used. In particular, Schreuder et al. (2014) used pleasant or unpleasant odor, Wackermann et al. (2014) used emotional sounds from the International Affective Digitized Sounds System; and Droit-Volet (2013) used aversive stimuli (acoustic stimuli producing a startle reflex). The first two papers (Schreuder et al., 2014; Wackermann et al., 2014) demonstrated that the judgment of time is systematically distorted with high-arousal/unpleasant emotional stimuli being judged longer than neutral stimuli or low-arousal/pleasant stimuli. In the same line, Zhang et al. (2014) showed that the presentation of Chinese words associated with fast (i.e., galloping, rapid) vs. slow (i.e., gradual) speed produced a lengthening effect in time bisection with the duration of the fast-speed words being judged to last longer than that of the slow-speed words. In addition, Droit-Volet (2013) showed that this lengthening effect is not specific to time judgment. Indeed, it can also be obtained in judgments of other quantities, i.e., number (a set of dots) and length (lines), when these quantities are presented sequentially. That is, the expectation of an aversive stimulus at the end of the quantity presentation made the participants to judge this quantity larger and longer than without aversive stimulus.

These psychological studies thus emphasize the sensitivity of time judgment to varying context. The findings demonstrate that the judgment of time is derived from a complex of context-related processes and cannot simply result from the accumulation of pulses (temporal units) emitted by an independent pacemaker-like system as suggested in the scalar expectancy theories (Gibbon et al., 1984). Indeed, the classical models of internal clock have some limits. They cannot straightforwardly account for numerous variations of time judgment in human beings such as those under an affective context or the phenomenon of increasing under-reproduction of intervals as duration is increased. The models on embodiment of time (Craig, 2009; Droit-Volet and Gil, 2009; Droit-Volet et al., 2013; Wittmann, 2013) assume that participants use bodily information (sensory-motor and/or interoceptive information) to judge time. Potentially, it can even be argued that the source of abstract pulses in fact are bodily/emotional information. In this perspective, Wackermann et al. (2014) demonstrated that the dual klepsydra model (DKM) can successfully model the emotion-related timing data. In their study, Pollatos et al. (2014) explored directly the role of biofeedback (heart-rate) in time estimation. They showed how the reproduction of intervals spanning half a second up to 40 s (starting and stopping intervals by button press) was to some extent phase locked with the cardiac cycle. Moreover, more accurate temporal reproduction was related to greater vagal control (a specific measure of heart-beat variability). The synchrony between performance in duration estimation and the (not consciously perceived) heart cycle is indicative of interoceptive (sympathovagal) processes underlying the perception of time in the seconds range. In a similar vein, Gouvêa et al. (2014) showed that the ability to estimate the passage of time is essential for adaptive behavior in complex environments. Animals developed highly reproducible behavioral sequences during the intervals being timed and used learned behavioral patterns to estimate the duration of time intervals. A prediction implied by this rationale is that of trial-to-trial variations in temporal estimation as dependent on behavioral fluctuations. Furthermore, Lacquaniti et al. (2014) provide a review on how the perception of physical events can be altered. For example, the assumption that things fall downward due to gravity and the relative context (right-side-up vs. upside-down environment) can have a profound effect on duration judgments. Also the form of the actor, i.e., animate (human-like) vs. inanimate (whirligig), has an influence. Based on these observations, it is argued that duration judgment is strongly linked with mechanisms used for motor control.

## Robotics

Insights gained from psychological studies on time perception can carry over to robotics, that is, to build robots and agents that can interact with humans in a more natural manner. In return, experimentation on robotic platforms can help direct future studies in psychology. Considering how time perception affects behavior execution and the timely accomplishment of tasks, De Kleijn et al. (2014) received inspiration from human cognition when discussing the temporal aspects of four behavioral principles important for robot control and for the seamless integration of autonomous artificial agents in human activities. These principles account for the integration of symbolic and sub-symbolic planning of action sequences, the integration of feed forward and feedback control, the clustering of complex actions into subcomponents, and the contextualization of action-control structures through goal representations. Considering the interaction among these principles is particularly important in order to equip robots with complex behaviors that robustly unfold over time.

On the other hand, Hinaut et al. (2014) show how robots acquire grammatical constructions and their temporal organization through human-robot interaction. The iCub robot used in their model is able to learn grammatical constructions such as predicate-argument representations through interactions with humans. The robot is also able to observe human-generated actions and generate natural language sentences to describe what it observed. Spatial relationship and temporal order of events have to be encoded in the grammatical constructions, which is achieved through the use of a recurrent neural network architecture (echo state network). Finally, they put the physical robotic system to test with human users, demonstrating learnability and generalization ability. An interesting dynamic emerged from the experiment, where human users adapted their interaction strategy to fit the level of competence of the robotic system. Investigating the timing of activation of neurons in the recurrent neural network relative to observed events and generated actions could lead to new insights on temporal processes in the brain.

## Neuroscience

Investigating neural underpinnings of timing and time-perception in the brain naturally leads to new complementary insights in this research topic. Interestingly, the two contributions from neuroscience in this issue deal with temporal duration in the context of prediction.

In everyday life, temporal intervals during which we have to wait are particularly intense regarding the experience of duration. Situations of temporal expectation of rewards are therefore well suited to investigate the underlying mechanisms of duration processing. Based on empirical work showing the dominant involvement of dopamine and the neural activity of dopaminergic areas in the brain, Vitay and Hamker (2014) propose a neuro-computational model of an afferent network as the neural substrate of temporal learning which includes the stages/processes of reward prediction, reward magnitude and reward delivery. Specifically, the model proposes that temporal contingencies between cues and rewards are learned through dopamine-modulated coincidence mechanisms in the nucleus accumbens.

The link of time interval prediction with consciousness is investigated in an original research article by Yoo et al. (2014) which focuses on the relationship between predictive dynamics and conscious states. Considering the dynamic temporal nature of consciousness, the authors test the hypothesis that predictable internal brain dynamics are correlated with conscious states. A computational study exploring publicly available EEG data from sleep and awake humans shows that conscious state dynamics are more predictable than unconscious (sleep) state dynamics. The results suggest an intricate relationship among prediction, consciousness, and time, with potential applications to time perception and neurorobotics.

## Conclusion

The endeavor of examining “time” as intrinsic part of embodied cognition is a particularly complex undertaking. The present collection of papers emphasizes the embodied nature of time perception in an attempt to strengthen the interaction between psychology and experimental work in the neurosciences on the one hand and computational modeling as well as robot cognition on the other hand. From this perspective, the papers published in this Research Topic “Toward embodied artificial cognition: TIME is on my side” aimed at addressing fundamental issues of time perception research and at the same time tried to include temporal aspects of various cognitive abilities in the so far “a-temporal domain” of robotics. Our wish and hope is that the papers published in the current research topic will increase the interdisciplinary scientific interest devoted to the study of time and will act as a research compass for future works in the field.

### Conflict of interest statement

The authors declare that the research was conducted in the absence of any commercial or financial relationships that could be construed as a potential conflict of interest.
